# The relative importance of maternal body mass index and glucose levels for prediction of large-for-gestational-age births

**DOI:** 10.1186/s12884-015-0722-x

**Published:** 2015-10-29

**Authors:** Kerstin Berntorp, Eva Anderberg, Rickard Claesson, Claes Ignell, Karin Källén

**Affiliations:** Department of Clinical Sciences Malmö, Lund University, Malmö, Sweden; Department of Endocrinology, Skåne University Hospital, Malmö, Sweden; Department of Clinical Sciences Lund, Lund University, Lund, Sweden; Department of Obstetrics and Gynecology, Office for Healthcare “Kryh”, Ystad, SE-27182 Sweden; Department of Obstetrics and Gynecology, Office for Healthcare “Sund”, Helsingborg, Sweden

**Keywords:** Body mass index, Gestational diabetes mellitus, Glucose levels, Large-for-gestational-age, Oral glucose tolerance test, Predicting risk

## Abstract

**Background:**

The risk of gestational diabetes mellitus (GDM) increases substantially with increasing maternal body mass index (BMI). The aim of the present study was to evaluate the relative importance of maternal BMI and glucose levels in prediction of large-for-gestational-age (LGA) births.

**Method:**

This observational cohort study was based on women giving birth in southern Sweden during the years 2003–2005. Information on 10 974 pregnancies was retrieved from a population-based perinatal register. A 75-g oral glucose tolerance test (OGTT) was performed in the 28 week of pregnancy for determination of the 2-h plasma glucose concentration. BMI was obtained during the first trimester. The dataset was divided into a development set and a validation set. Using the development set, multiple logistic regression analysis was used to identify maternal characteristics associated with LGA. The prediction of LGA was assessed by receiver-operating characteristic (ROC) curves, with LGA defined as birth weight > +2 standard deviations of the mean.

**Results:**

In the final multivariable model including BMI, 2-h glucose level and maternal demographics, the factor most strongly associated with LGA was BMI (odds ratio 1.1, 95 % confidence interval [CI] 1.08–1.30). Based on the total dataset, the area under the ROC curve (AUC) of 2-h glucose level to predict LGA was 0.54 (95 % CI 0.48–0.60), indicating poor performance. Using the validation database, the AUC for the final multiple model was 0.69 (95 % CI 0.66–0.72), which was identical to the AUC retrieved from a model not including 2-h glucose (0.69, 95 % CI 0.66–0.72), and larger than from a model including 2-h glucose but not BMI (0.63, 95 % CI 0.60–0.67).

**Conclusions:**

Both the 2-h glucose level of the OGTT and maternal BMI had a significant effect on the risk of LGA births, but the relative contribution was higher for BMI. The findings highlight the importance of concentrating on healthy body weight in pregnant women and closer monitoring of weight during pregnancy as a strategy for reducing the risk of excessive fetal growth.

## Background

Obesity is an increasing health problem, and affects up to one-third of women of reproductive age in the western world [1]. The risk of gestational diabetes mellitus (GDM) increases substantially with increasing maternal body mass index (BMI) [2]. Moreover, GDM and maternal obesity are independently associated with adverse neonatal outcomes, in particular macrosomia and large-for-gestational-age (LGA) births [3–5], which in turn increase the risk of complications in both the mother and the newborn [6]. For the mother this includes prolonged labour, perineal lacerations, uterine atonia, abnormal haemorrhage and caesarean section [6, 7]. Neonatal complications consist of birth trauma associated with shoulder dystocia, hypoglycaemia, respiratory distress and may also result in impairment to health later in life [6, 7]. Antenatal detection of large fetuses makes it possible to intervene by induction of labour or caesarean section, thereby preventing the birth of macrosomic newborns or complications associated with vaginal delivery of large babies. Surkan et al. reported an unadjusted increase in LGA births in Sweden of 23 % over the years 1992–2001. The increasing trend could mainly be explained by concurrent increases in maternal BMI and decreases in maternal smoking [8]. The prevalence of maternal smoking has declined continuously in Sweden during the last decades with an annual change of 7.2 % between 2000 and 2008 [9].

Universal screening for GDM by an oral glucose tolerance test (OGTT) has been performed at the general antenatal clinics in southern Sweden since 1995. The screening program is well implemented and has previously shown high adherence, with 93 % of eligible women being screened [10]. During the years 2003–2005, pregnant women representing different glucose categories according to the 2-h glucose level of the OGTT were invited to take part in a follow-up program, the Mamma Study. The pregnancy outcomes of the participating women have been reported previously, indicating that even limited degrees of maternal hyperglycemia affect the outcome and increase the risk of LGA births [11]. During the period of recruitment to the Mamma Study, a large number of test results from the antenatal clinics were made available. These form the basis of the present study. The purpose was to evaluate the relative importance of BMI and glucose levels in prediction of LGA births in a large sample of the pregnant population, also taking other risk factors into account by adding information on maternal characteristics.

## Methods

### GDM screening

The screening program for GDM in southern Sweden has been described in detail previously [11]. Briefly, a 75-g OGTT is offered to all women in the 28 week of gestation, and is done after overnight fasting at their local antenatal clinic. The diagnostic criteria for GDM are a simplification of those recommended by the European Association for the Study of Diabetes, omitting the initial fasting glucose sample and defining GDM as a 2-h capillary blood glucose concentration of ≥ 9.0 mmol/L [12]. In 2004, routine glucose measurements in Sweden were switched from blood glucose measurements to plasma glucose measurements, and a transformation factor of 1.11 was agreed on [13], resulting in a 2-h threshold value of 10.0 mmol/L for capillary plasma glucose to define GDM. The HemoCue blood glucose system (HemoCue AB, Ängelholm, Sweden) is used to obtain immediate analysis of glucose concentrations. If 2-h capillary plasma glucose concentration is 8.9–9.9 mmol/L, indicating gestational impaired glucose tolerance (IGT), the OGTT is repeated within a week. Normal glucose tolerance during pregnancy is defined as a 2-h capillary plasma glucose concentration < 8.9 mmol/L.

### Study population

Recruitment to the Mamma Study took place in 2003–2005, and involved four of the five delivery departments in the county of Skåne in southern Sweden; details have been described previously [11]. During the recruitment period, OGTT results from the local antenatal clinics were sent to the study coordinator (EA), enabling identification of the test results of women who consented to be enrolled; it also ensured correct sampling technique [10]. Initially, 11 976 OGTT results in total were reported. If a woman had repeated pregnancies during the period, only the first one was included. Likewise, if a repeat OGTT was performed, only the first one was included.

Participating women received standard obstetric care as long as their OGTT values were normal. Women diagnosed with GDM were transferred to specialist antenatal care and had regular contact with a diabetologist. They were given advice on diet and physical exercise, and they were closely monitored through self-testing of blood glucose. If treatment goals for blood glucose were not achieved, insulin treatment was added. Women diagnosed with gestational IGT were given advice on diet and physical exercise, but followed the routine pregnancy program, unless a repeat OGTT was diagnostic of GDM.

The study was carried out in accordance with the Declaration of Helsinki. Written informed consent was obtained from all participants and the study protocol was approved by the Ethics Committee of Lund University (LU 259–00).

### Perinatal Revision South (PRS)

Population-based information was retrieved from the regional perinatal database, Perinatal Revision South (PRS), which was established in 1995 for quality assurance in perinatal care in the southern region of Sweden [14]. The PRS is based on approximately 18 000 annual births, and is compiled from data reported by all delivery and neonatal units in the region. The maternal pregnancy characteristics used as exposure variables were maternal age at delivery, parity, BMI, maternal height and maternal smoking. Information about BMI (kg/m^2^) was based on weight and height measured at the first prenatal visit in the first trimester. Gestational age was estimated from expected date of parturition according to ultrasound in the first half of gestation. LGA births, small-for-gestational-age (SGA) births and adequate-for-gestational-age (AGA) births were defined as birth weight greater than +2 standard deviations (SD), less than −2 SD and between −2 SD and +2 SD of the expected birth weight for gestational age and gender, respectively, according to the Swedish reference curve for fetal growth [15]. Of the 11 976 OGTT results, information in the PRS was available for a total of 11 016 pregnancies. When we evaluated the risk factors for LGA, infants with unavailable LGA information were excluded, and this restricted dataset was the basis of the present evaluation (n = 10 974). The dataset was divided into two parts, with every second woman belonging to the development dataset or the validation dataset.

### Statistical analysis

Differences in glucose levels between groups were assessed using the Kruskal-Wallis test.

Chi-squared tests were performed to test possible differences between the datasets regarding maternal and infant characteristics (i.e. the development dataset and the validation dataset). The correlation between maternal BMI and 2-h glucose levels was estimated using the Pearson rho, and the linear relationship was estimated using a simple linear regression.

The prediction model for LGA was developed on the development dataset using univariate and multivariable logistic regression analyses. The variables tested were: maternal age (in years; continuous variable), parity 1, parity ≥ 4 (with parity 2–3 as reference), maternal smoking (yes/no), maternal BMI (in kg/m^2^; continuous), maternal height (in cm; continuous), and glucose levels (in mmol/L; continuous). Models including class variables or second-degree polynomials were tested, but were abandoned as they performed worse than the models including the linear, continuous variables mentioned. Variables with a crude *p*-value of < 0.05 in their association with LGA in the univariate model were entered into a multiple model, and variables with a *p*-value of < 0.05 in the multiple model were entered into the final multiple model. A two-sided *p*-value of less than 0.05 was considered statistically significant.

The results obtained from the final multiple model, and two other models for comparison, were applied to the validation dataset. The performance of each model was evaluated by studying the area under the receiver-operating characteristics (ROC) curve (AUC). The variance of each AUC was computed using the method proposed by DeLong et al. [16].

All statistical analyses were performed using Gauss (Gauss™; Aptec Systems Inc., Maple Valley, WA, USA; http://www.aptech.com).

## Results

**Fig. 1:**
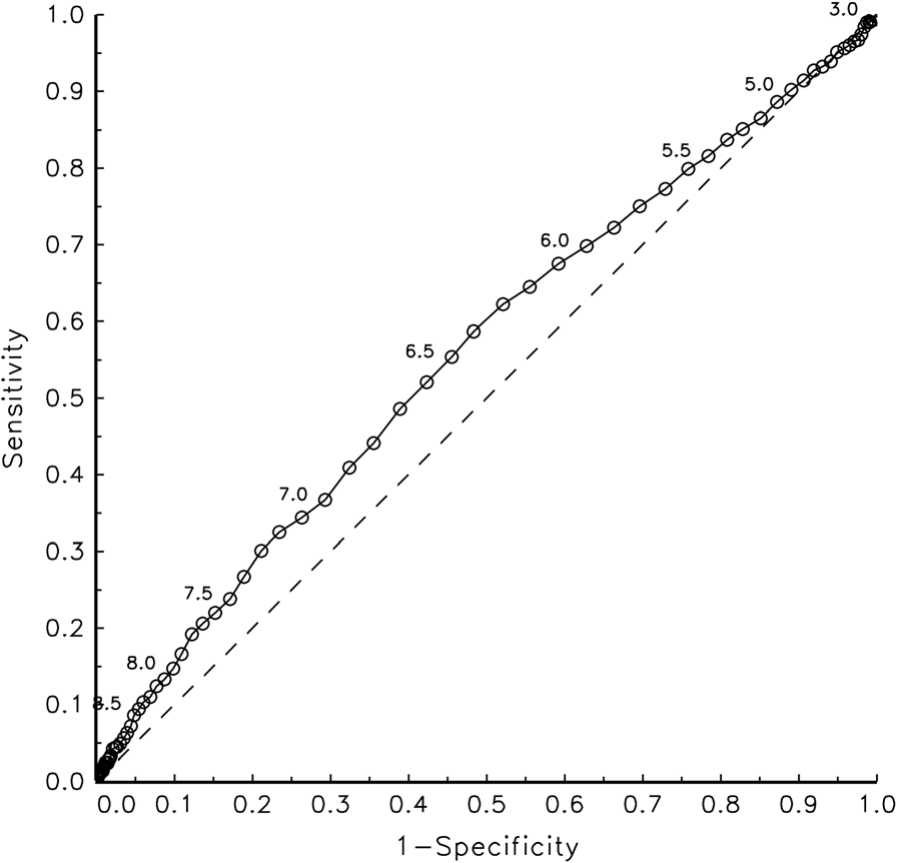
The overall ability of glucose to predict large-for-gestational-age births

The frequency of maternal and infant characteristics according to glucose quartile and the corresponding mean 2-h plasma glucose levels are given in Table 1. Of the 2777 women with glucose levels in the upper quartile, 120 (1.1 % of all women) fulfilled the glucose threshold for GDM (2-h plasma glucose concentration ≥ 10.0 mmol/L) and 301 (2.7 % of all women) fulfilled the glucose threshold for gestational IGT (2-h plasma glucose concentration 8.9–9.9 mmol/L). A linear regression analysis showed a weak, albeit statistically significant, linear association between maternal BMI and glucose levels (increase of 2-h plasma glucose per each BMI-unit: 0.022; 95 % CI 0.017–0.028), with a statistically significant, but weak correlation coefficient (Pearson rho: 0.074; 95 % CI: 0.056–0.093). A ROC curve based on the total dataset revealed that the ability of the 2-h glucose levels to predict LGA births was poor; AUC was 0.54 (95 % CI 0.48–0.60) (Fig. 1). Furthermore, there was no apparent natural cutoff point above which there would be an increased risk of LGA in the infant.

**Table 1:** Maternal and infant characteristics according to glucose quartiles, and the corresponding 2-h plasma glucose level

Glucose quartiles (mmol/L)	<5.7		5.7–6.4		6.5–7.2		>7.20		2-h Glucose (mmol/L)		*p* ^a^
	n	%	n	%	n	%	n	%	mean	95 % CI	
Total	2637	23.9	2783	25.3	2819	25.6	2777	25.2			
Maternal age, years											<0.001
<20	80	32.5	62	25.2	63	25.6	41	16.7	6.2	6.1–6.4	
20–34	2148	24.2	2288	25.8	2264	25.5	2180	24.5	6.5	6.4–6.5	
≥35	409	21.6	433	22.9	492	26.0	556	29.4	6.6	6.6–6.7	
Parity											0.09
1	128	23.8	134	24.9	141	26.2	135	25.1	6.5	6.4–6.5	
2–3	119	24.1	128	26.0	124	25.2	122	24.7	6.5	6.4–6.5	
≥4	16	24.1	15	22.5	15	23.4	20	30.0	6.6	6.5–6.7	
Smoker											<0.001
No	2220	23.4	2408	25.4	2430	25.6	2424	25.6	6.5	6.5–6.5	
Yes	341	27.2	309	24.6	333	26.6	271	21.6	6.3	6.3–6.4	
Maternal BMI, kg/m^2^											<0.001
<18.5	50	25.6	50	25.6	50	25.6	45	23.1	6.4	6.3–6.6	
18.5–24	1496	25.1	1569	26.3	1542	25.9	1351	22.7	6.4	6.4–6.4	
25.0–29.9	585	22.0	641	24.1	687	25.9	743	28.0	6.6	6.5–6.6	
30–34.9	182	20.8	187	21.4	223	25.5	281	32.2	6.6	6.6–6.7	
≥35	83	20.1	103	25.0	93	22.6	133	32.3	6.8	6.7–6.9	
Gestational age, weeks											0.006
<37	117	20.0	148	25.3	153	26.2	167	28.5	6.7	6.5–6.8	
37–41 + 6	2345	24.0	2472	25.3	2502	25.6	2452	25.1	6.5	6.4–6.5	
≥42 + 0	175	26.5	163	24.7	164	24.8	158	23.9	6.4	6.3–6.5	
Weight for gestational age											<0.001
SGA	69	23.2	80	26.9	68	22.9	80	26.9	6.5	6.4–6.7	
AGA	2446	24.2	2577	25.5	2578	25.6	2495	24.7	6.5	6.4–6.5	
LGA	115	20.1	110	19.2	156	27.3	191	33.4	6.7	6.6–6.9	
Infant gender											0.9
Male	1407	24.5	1415	24.5	1437	25.0	1479	25.8	6.5	6.4–6.5	
Female	1228	23.4	1359	25.8	1379	26.2	1292	24.6	6.5	6.5–6.5	

*BMI* body mass index, *CI* confidence interval, *SGA* small-for-gestational-age, *AGA* adequate-for-gestational-age, *LGA* large-for-gestational-age

^a^ *p*-values obtained by non-parametric tests (Kruskal-Wallis) for difference in glucose level between the specified groups

The maternal and infant characteristics of the development and validation groups are given in Table 2. The demographic characteristics of the groups were similar, but by chance there were significantly more women with BMI above 35, and SGA infants, in the development dataset than in the validation dataset.

**Table 2:** Demographic characteristics of development sample and validation sample groups

Characteristic	Development sample (n = 5487)		Validation sample (n = 5487)		*p* ^*a*^
Maternal age, years	29.7	5.1	29.6	5.1	0.88
< 20	121	(2.2)	125	(2.3)	0.80
20–34	4415	(80.5)	4426	(80.7)	0.79
≥ 35	951	(17.3)	936	(17.1)	0.71
Parity					
1	2688	(49.0)	2681	(48.9)	0.90
2–3	2463	(44.9)	2465	(44.9)	0.97
≥ 4	336	(6.1)	341	(6.2)	0.84
Smoker					
No	4727	(86.1)	4722	(86.1)	0.89
Yes	625	(11.4)	623	(11.4)	0.96
Maternal BMI, kg/m^2^	24.9	4.5	24.7	4.3	0.089
< 18.5	102	(1.9)	92	(1.7)	0.47
18.5–24	2928	(53.4)	3015	(54.9)	0.095
25.0–29.9	1303	(23.7)	1343	(24.5)	0.37
30–34.9	440	(8.0)	424	(7.7)	0.57
≥ 35	236	(4.3)	175	(3.2)	0.002
Gestational age, weeks	39.7	1.7	39.7	1.7	0.62
< 37	304	(5.5)	281	(5.1)	0.33
37–41 + 6	4875	(88.8)	4889	(89.1)	0.67
≥ 42 + 0	308	(5.6)	317	(5.8)	0.71
Weight for gestational age					
SGA	166	(3.0)	131	(2.4)	0.04
AGA	5044	(91.9)	5061	(92.2)	0.58
LGA	277	(5.0)	295	(5.4)	0.44
Infant gender					
Male	2839	(51.7)	2888	(52.6)	0.35
Female	2648	(48.3)	2599	(47.4)	0.35

Both groups contain only information where all information was available. Data are n (%) or mean (SD)

*AGA* adequate for gestational age, *BMI* body mass index, *LGA* large-for-gestational-age, *SGA* small-for-gestational-age

^a^ *p*-values obtained by chi-squared test (1 DF) for class variables and by Mann-Whitney *U*-test for continuous data

Table 3 shows the odds ratios for LGA obtained from univariate and multiple logistic regression analyses based on the development sample. In the univariate analysis, all the factors evaluated except height (*p* = 0.0831, not shown) and parity ≥ 4 were significantly associated with LGA. In the first multiple model (including all the significant variables), all variables except maternal age remained significant. In the final multiple model, excluding maternal age, the factor most strongly associated with LGA was BMI (*p* = 2.6 × 10^−19^), accounting for 4.3 % of the variance in the univariate setting (R^2^ = 0.043). Using the validation database, the AUC for the final multiple model was 0.69 (95 % CI 0.66–0.72), which was identical to the AUC retrieved from a model not including 2-h glucose (AUC 0.69 [95 % CI 0.66–0.72]), and larger than from a model including 2-h glucose but not BMI (AUC 0.63 [95 % CI 0.60–0.67]).

**Table 3:** Risk factors for large-for-gestational-age infants in development sample, using univariate and multiple logistic regression analysis

	Univariate model		Multiple model		Final multiple model		
Risk factor	OR	*p*	OR	*p*	OR	95 % CI	*p*
Maternal age (per 1-year increase)	1.04	0.005	1.01	0.677			
Body mass index (per 1-step increase)	1.11	<0.001	1.10	<0.001	1.10	1.08–1.13	<0.001
2-h glucose (per 1 mmol increase)	1.12	0.003	1.09	0.033	1.09	1.01–1.18	0.028
Smoker	0.31	<0.001	0.29	<0.001	0.29	0.16–0.52	<0.001
Parity 1	0.48	<0.001	0.52	<0.001	0.51	0.40–0,67	<0.001
Parity ≥ 4	0.98	0.917					

Multiple model included variables with *p* < 0.05 in univariate model. Final multiple model included variables with *p* < 0.05 in primary multiple model

*OR* odds ratio, *CI* confidence interval

**Fig. 2:**
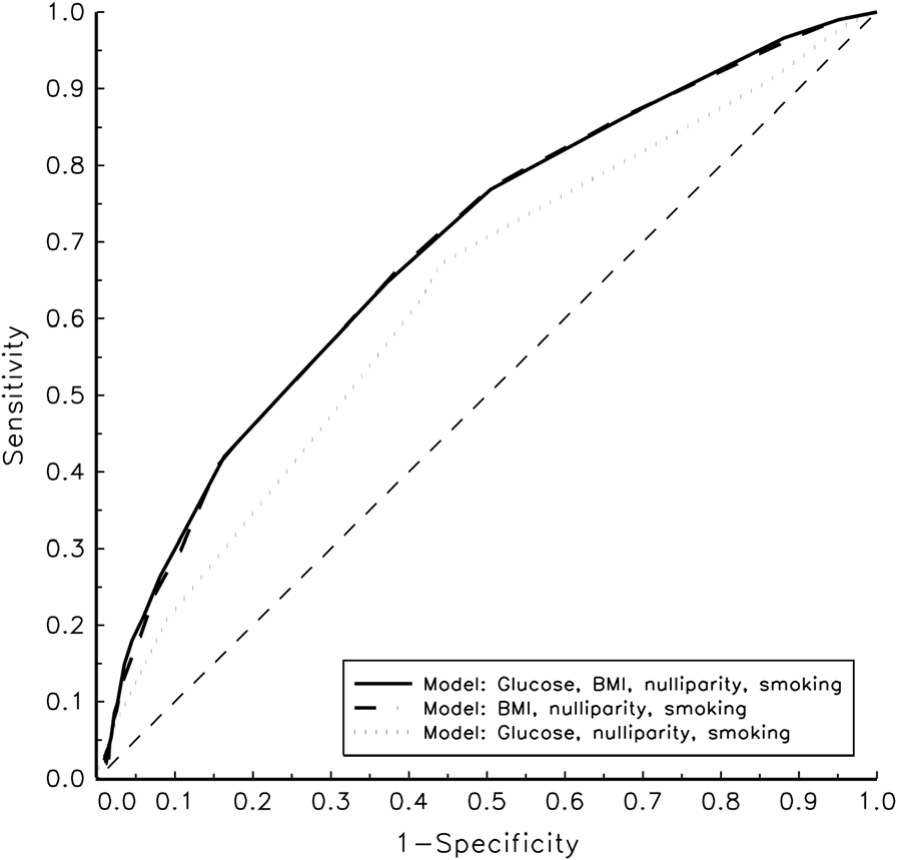
ROC curves obtained after application of the three prediction models based on the validation data

The overall abilities of the three models developed in predicting LGA in the validation sample were illustrated using ROC curves (Fig. 2). The figure clearly shows that the ROC curve based on the model including BMI, nulliparity and maternal smoking was identical to that based on the model in which glucose levels were also added, whereas the performance of the model that included glucose levels but not BMI was considerably poorer.

## Discussion

The main findings of the present study were that both the 2-h glucose level of the OGTT and maternal BMI had a significant effect on the risk of delivering an LGA neonate. However, the relative contribution was much higher for BMI, even when taking other risk factors into account. The overall ability of the developed model to predict LGA in the validation sample was satisfactory, but was identical to that of a model that did not include the 2-h glucose level.

The lack of internationally uniform diagnostic criteria for GDM, and the lack of agreement regarding what glucose levels should define normal glucose tolerance during pregnancy, hampers comparisons between studies [17]. Similar to our study, using the 2-h threshold of the WHO 1999 criteria to define normal glucose tolerance during pregnancy [18], a Danish study investigated the relationship between pregnancy outcome and pregnancy overweight or obesity in 2459 women with normal glucose tolerance during pregnancy [19]. After adjustment for various risk indicators, including the 2-h glucose value during the OGTT, they found a progressively increased risk of LGA births in overweight and obese women. However, they did not evaluate the corresponding effect of glucose levels when controlling for BMI and other risk indicators. It should be noted that the LGA was defined as birth weight above the ninetieth percentile for the reference population, which differed from the one used in the current study (approximately equivalent to the 97.5th percentile).

Based on the ROC curve of the total dataset, we found no apparent natural cutoff point above which there would be an increased risk of having an LGA infant. This is in line with the Hyperglycemia and Adverse Pregnancy Outcomes (HAPO) Study, which showed that maternal hyperglycemia is associated with perinatal risk in a linear way, with no obvious threshold [20]. In a post hoc analysis using the International Association of Diabetes and Pregnancy Study Groups (IADPSG) criteria for GDM [21], OR for birth weight greater than the ninetieth percentile was somewhat higher in non-obese GDM women (2.19, 95 % CI 1.93–2.47) than in obese non-GDM women (1.73, 95 % CI 1.50–2.0) relative to non-obese non-GDM women, controlling for other potential risk factors [4]. Whereas all other guidelines for the diagnosis of GDM are more or less based on arbitrary statistics, the IADPSG criteria are for the first time based on perinatal outcomes [22]. According to these criteria, at least one of the fasting, 1-h or 2-h venous plasma glucose thresholds during a 75-g OGTT (5.1, 10.0 or 8.5 mmol/L, respectively) must be equalled or exceeded to make a GDM diagnosis. Use of the individual glucose thresholds fasting, 1-h and 2 h identified 55, 55 and 38 %, respectively, of the total HAPO cohort [23]. Although it is not regarded as a diagnostic standard [21], capillary glucose samples are widely used for diagnostic purposes in Sweden. According to a recently presented conversion algorithm, the capillary 2-h threshold value of 10.0 mmol/L—used in most parts of Sweden to define GDM [24] —coincides with the venous 2-h threshold value proposed by the IADPSG [25]. From this, it is obvious that the simplified method, omitting the initial fasting glucose sample during the OGTT, is not optimal for prediction of gestational weight of the newborn.

The main strength of the present study was the uniform diagnostic procedure for GDM, based on universal screening with a 75-g OGTT, enabling identification of a rather large cohort of women with test results over the entire glucose scale. In our previous report from the Mamma Study, suggesting that moderately increased glucose levels may also affect pregnancy outcome, adjustments for BMI were not performed because the information was not available at the time [11]. In light of the present findings, it is reasonable to assume that adjustment for BMI would have attenuated the results to some extent. However, as the current study showed that the correlation between BMI and glucose levels was rather weak, it is not likely that the results would be heavily influenced from BMI. Furthermore, since the control group in the previous study included only one twenty-fourth of consenting women with normal glucose tolerance during pregnancy, the material did not allow prediction analysis. We have previously shown that maternal characteristics such as age, parity and smoking—in addition to BMI and maternal glucose status—influence fetal growth during the last trimester [26]. The logistic regression modelling identified the independent variables available from the register that are important and can help in the prediction of LGA births.

It could be argued that women with glucose levels in the IGT range and above, receiving some kind of advice or treatment during pregnancy may have biased the results. However, it is likely that the risk of LGA births would have increased even more if these women had not been taken care of. Another possible weakness of the study was the lack of information regarding ethnicity. Disparities in ethnicity/race may affect the impact of obesity and glucose status on perinatal outcomes [27–29]. Furthermore, the prediction model might have been more powerful if maternal weight gain during pregnancy had been considered. Both maternal pre-pregnancy obesity and excessive gestational weight gain lead to increased risk of adverse pregnancy outcomes, including LGA. Overall, the associations between maternal pre-pregnancy obesity and adverse pregnancy outcomes appear to be stronger than those between excessive gestational weight gain and adverse pregnancy outcomes [30], although some studies have indicated that gestational weight gain is of greater importance [5, 31].

## Conclusions

Based on the present material, we conclude that maternal BMI had a greater impact on the prediction of LGA birth than the 2-h glucose level of the OGTT. The overall performance of the full prediction model, also taking other risk factors into account, was satisfactory. The data highlight the importance of targeting healthy body weight in pregnant women and closer monitoring of weight during pregnancy as a strategy for reducing the risk of excessive fetal growth. A number of intervention trials have been published and show heterogeneous results in efficacy in reducing excess gestational weight gain [32, 33]. Adequately powered intervention studies are needed to provide evidence-based guidelines to facilitate pregnant women in achieving weight gain within recommended limits with the aim to reduce neonatal adiposity.

## Abbreviations

AGA: adequate-for-gestational-age
AUC: area under the ROC curve
BMI: body mass index
CI: confidence interval
GDM: gestational diabetes mellitus
HAPO: Hyperglycemia and Adverse Pregnancy Outcome
IADPSG: International Association of Diabetes and Pregnancy Study Groups
IGT: impaired glucose tolerance
LGA: large-for-gestational-age
OGTT: oral glucose tolerance test
OR: odds ratio
PRS: Perinatal Revision South
ROC curve: receiver-operating characteristic curve
SD: standard deviation
SGA: small-for-gestational-age
WHO: World Health Organization
